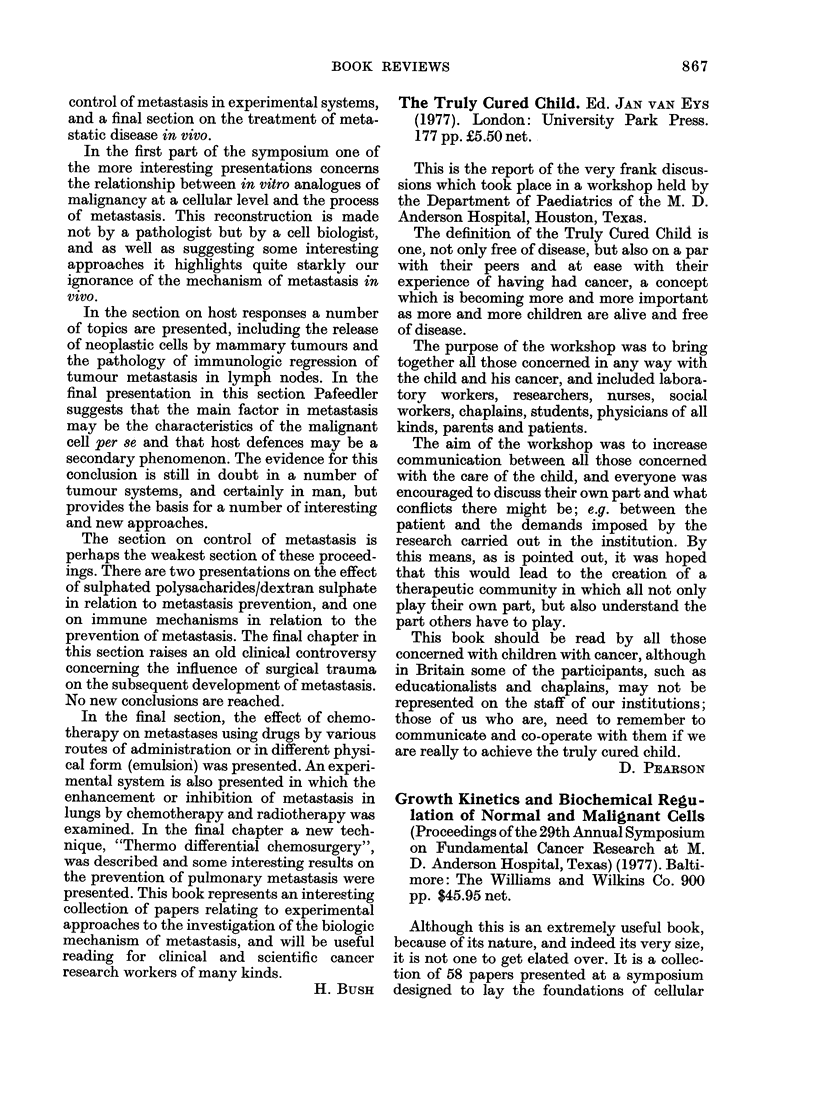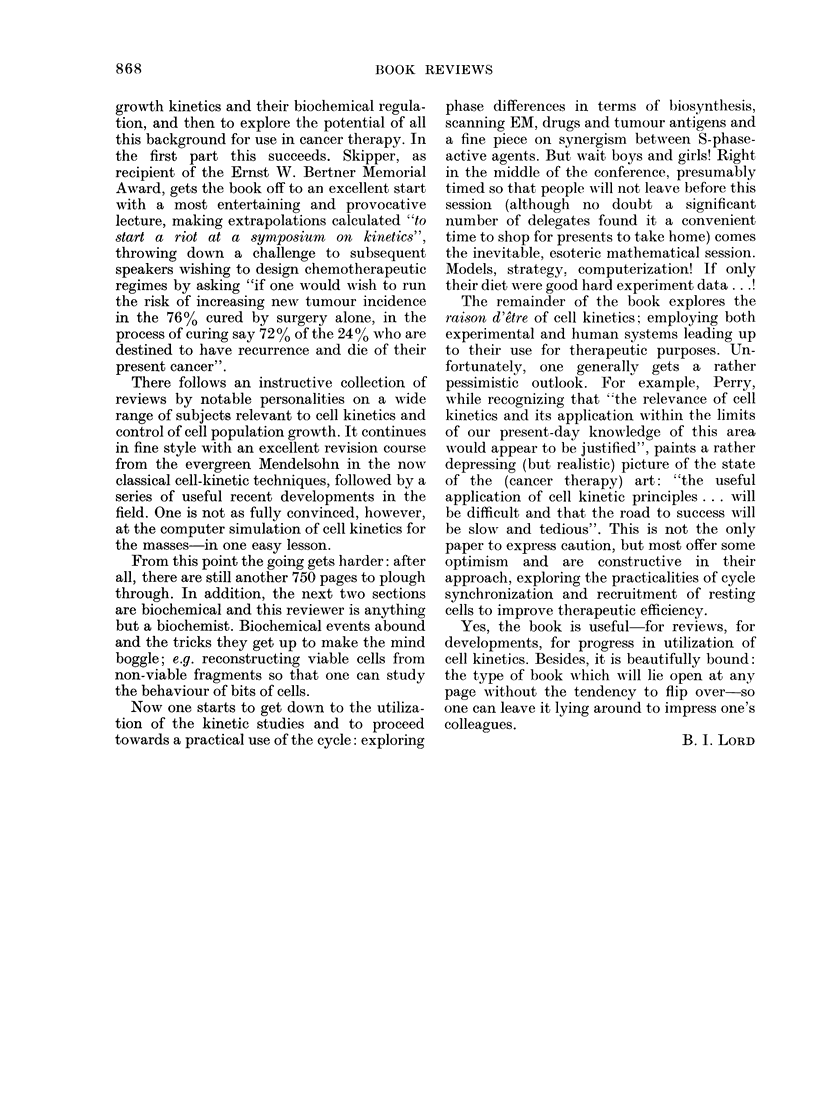# Growth Kinetics and Biochemical Regulation of Normal and Malignant Cells

**Published:** 1978-05

**Authors:** B. I. Lord


**Growth Kinetics and Biochemical Regulation of Normal and Malignant Cells** (Proceedings of the 29th Annual Symposium on Fundamental Cancer Research at M. D. Anderson Hospital, Texas) (1977). Baltimore: The Williams and Wilkins Co. 900 pp. $45.95 net.

Although this is an extremely useful book, because of its nature, and indeed its very size, it is not one to get elated over. It is a collection of 58 papers presented at a symposium designed to lay the foundations of cellular growth kinetics and their biochemical regulation, and then to explore the potential of all this background for use in cancer therapy. In the first part this succeeds. Skipper, as recipient of the Ernst W. Bertner Memorial Award, gets the book off to an excellent start with a most entertaining and provocative lecture, making extrapolations calculated "*to start a riot at a symposium on kinetics*", throwing down a challenge to subsequent speakers wishing to design chemotherapeutic regimes by asking "if one would wish to run the risk of increasing new tumour incidence in the 76% cured by surgery alone, in the process of curing say 72% of the 24% who are destined to have recurrence and die of their present cancer".

There follows an instructive collection of reviews by notable personalities on a wide range of subjects relevant to cell kinetics and control of cell population growth. It continues in fine style with an excellent revision course from the evergreen Mendelsohn in the now classical cell-kinetic techniques, followed by a series of useful recent developments in the field. One is not as fully convinced, however, at the computer simulation of cell kinetics for the masses—in one easy lesson.

From this point the going gets harder: after all, there are still another 750 pages to plough through. In addition, the next two sections are biochemical and this reviewer is anything but a biochemist. Biochemical events abound and the tricks they get up to make the mind boggle; *e.g.* reconstructing viable cells from non-viable fragments so that one can study the behaviour of bits of cells.

Now one starts to get down to the utilization of the kinetic studies and to proceed towards a practical use of the cycle: exploring phase differences in terms of biosynthesis, scanning EM, drugs and tumour antigens and a fine piece on synergism between S-phase-active agents. But wait boys and girls! Right in the middle of the conference, presumably timed so that people will not leave before this session (although no doubt a significant number of delegates found it a convenient time to shop for presents to take home) comes the inevitable, esoteric mathematical session. Models, strategy, computerization! If only their diet were good hard experiment data . . .!

The remainder of the book explores the *raison d'être* of cell kinetics; employing both experimental and human systems leading up to their use for therapeutic purposes. Unfortunately, one generally gets a rather pessimistic outlook. For example, Perry, while recognizing that "the relevance of cell kinetics and its application within the limits of our present-day knowledge of this area would appear to be justified", paints a rather depressing (but realistic) picture of the state of the (cancer therapy) art: "the useful application of cell kinetic principles . . . will be difficult and that the road to success will be slow and tedious". This is not the only paper to express caution, but most offer some optimism and are constructive in their approach, exploring the practicalities of cycle synchronization and recruitment of resting cells to improve therapeutic efficiency.

Yes, the book is useful—for reviews, for developments, for progress in utilization of cell kinetics. Besides, it is beautifully bound: the type of book which will lie open at any page without the tendency to flip over—so one can leave it lying around to impress one's colleagues.

B. I. Lord